# “One more time”: time loops as a tool to investigate folk conceptions of moral responsibility and human agency

**DOI:** 10.1007/s11229-023-04245-9

**Published:** 2023-08-29

**Authors:** Thibaut Giraud, Maicol Neves Leal, Florian Cova

**Affiliations:** 1 Institut Jean Nicod, Paris, France; 2 Literature & Philosophy Department, University of Geneva, Geneva, Switzerland; 3 Philosophy Department & Swiss Center for Affective Sciences, University of Geneva, Geneva, Switzerland

**Keywords:** Determinism, Experimental philosophy, Free will, Moral responsibility, Time travel

## Abstract

In the past 20 years, experimental philosophers have investigated folk intuitions about free will and moral responsibility, and their compatibility with determinism. To determine whether laypeople are “natural compatibilists” or “natural incompatibilists”, they have used vignettes describing agents living in deterministic universes. However, later research has suggested that participants’ answers to these studies are plagued with comprehension errors: either people fail to really accept that these universes are deterministic, or they confuse determinism with something else. This had led certain experimenters to conclude that maybe folk intuitions about the compatibility of free will with determinism could not be empirically investigated. Here, we propose that we should refrain from embracing this pessimistic conclusion, as scenarios involving time loops might allow experiments to bypass most of these methodological issues. Indeed, scenarios involving time loops belong both to the philosophical literature on free will and to popular culture. As such, they might constitute a bridge between the two worlds. We present the results of five studies using time loops to investigate people’s intuitions about determinism, free will and moral responsibility. The results of these studies allow us to reach two conclusions. The first is that, when people are introduced to determinism through time loops, they do seem to understand what determinism entails. The second is that, at least in the context of time loops, people do not seem to consider determinism to be incompatible with free will and moral responsibility.

**Supplementary Information:**

The online version contains supplementary material available at 10.1007/s11229-023-04245-9.

## Methodological issues in experimental philosophy of free will

Determinism is the claim that, given the past and the laws of nature, everything that happens in the world had to happen. What the truth of determinism would entail for human agency has been the topic of heated debates among philosophers (Fischer et al., 2009). Compatibilists consider that determinism is compatible with both free will and moral responsibility, while incompatibilists consider that determinism would prevent free will and moral responsibility. To argue for their position, both sides appeal to common sense intuitions about free will and moral responsibility. These appeals to intuition can be appeals to intuitions about general, abstract principles such as the Principle of Alternate Possibilities (Widerker & McKenna, 2006) or the Transfer of Non-Responsibility (Capes, 2016; van Inwagen, 1983). But they can also appeal to intuitions about particular cases such as Frankfurt-style cases (Cova, 2014; Frankfurt, 1969) or Manipulation cases (Mele, 2019; Pereboom, 1995).

This reliance on common sense intuitions has drawn the attention of experimental philosophers who decided to empirically investigate folk intuitions about the compatibility of determinism with free will, moral responsibility, and human agency (for an overview, see Feltz, 2017; Cova, forthcoming-a). Thus, for the past 20 years, experimental philosophers have studied how people without expertise or prior training in philosophy felt about the relevance of determinism to free will and moral responsibility. Some of these studies have borne on specific intuitions such as intuitions about Frankfurt-style cases (Cova, 2017; Miller & Feltz, 2011) or Manipulation cases (Cova, 2022; Feltz, 2013; Sripada, 2012). However, most of them have investigated whether people directly perceive determinism to be incompatible with free will and moral responsibility (Nahmias et al., 2005, 2006). These studies have thus contributed to the rise of a second, parallel debate: whether laypeople are “natural compatibilists” or “natural incompatibilists” (Feltz et al., 2009).

As we mentioned earlier, it has been almost 20 years since experimental philosophers began investigating this question (see Nichols, 2004). One might thus expect them to have reached a consensus answer: how hard can it be to assess whether people consider determinism to be incompatible with free will and moral responsibility? The answer is: pretty hard, actually.

As Nahmias and colleagues (2005, 2006) point out, one cannot just ask people whether they consider determinism to be compatible with free will and moral responsibility. The reason is that “determinism” is a technical term and that most people spontaneously interpret it as meaning the opposite of free will. Thus, experimental philosophers need to find a way to convey the technical meaning of determinism to participants. In a series of seminal studies on the question, Nahmias and colleagues (2005, 2006) decided to use vignettes describing deterministic universes and to ask participants whether agents living in these universes can be morally responsible for their actions. Here is an example:

*Rollback* - Imagine there is a universe that is recreated over and over again, starting from the exact same initial conditions and with all the same laws of nature. In this universe the same conditions and the same laws of nature produce the exact same outcomes, so that every single time the universe is re-created, everything must happen the exact same way. For instance, in this universe a person named Jill decides to steal a necklace at a particular time, and every time the universe is re-created, Jill decides to steal the necklace at that time.

Participants were asked to judge whether Jill decided to steal the necklace of her own free will and whether “it would be fair to hold her morally responsible (that is, blame her) for her decision to steal the necklace”. 66% judged that Jill acted of her own free will, and 77% judged her to be morally responsible, suggesting that roughly two thirds of participants did not see any incompatibility between free will and determinism. Other vignettes yielded similar results.

On the basis of such results, we might be tempted to conclude that most people are “natural compatibilists”. However, things are not this simple. In a later study, Nichols and Knobe (2007) sought to compare participants’ intuitions about *abstract* and *concrete* cases. They first presented their participants with a description of two universes: a deterministic universe (Universe A) and an indeterministic universe (Universe B). Here is how it went:

Imagine a universe (Universe A) in which everything that happens is completely caused by whatever happened before it. This is true from the very beginning of the universe, so what happened in the beginning of the universe caused what happened next, and so on right up until the present. For example, one day John decided to have French Fries at lunch. Like everything else, this decision was completely caused by what happened before it. So, if everything in this universe was exactly the same up until John made his decision, then it *had to happen* that John would decide to have French Fries.

Now imagine a universe (Universe B) in which *almost* everything that happens is completely caused by whatever happened before it. The one exception is human decision making. For example, one day Mary decided to have French Fries at lunch. Since a person’s decision in this universe is not completely caused by what happened before it, even if everything in the universe was exactly the same up until Mary made her decision, it *did ****not**** have to happen* that Mary would decide to have French Fries. She could have decided to have something different.

The key difference, then, is that in Universe A every decision is completely caused by what happened before the decision-given the past, each decision *has to happen* the way that it does. By contrast, in Universe B, decisions are not completely caused by the past, and each human decision *does ****not**** have to happen* the way that it does.

Half of participants were then presented with the *concrete* case:

In Universe A, a man named Bill has become attracted to his secretary, and he decides that the only way to be with her is to kill his wife and 3 children. He knows that it is impossible to escape from his house in the event of a fire. Before he leaves on a business trip, he sets up a device in his basement that burns down the house and kills his family.

Is Bill fully morally responsible for killing his wife and children? (YES/NO)

In this case, 72% answered that Bill was fully morally responsible for killing his wife and children, even though he lived in a deterministic universe. This is in line with the results that Nahmias and colleagues obtained for the *Rollback* case.

However, the other half of participants was presented with the *abstract* case:

In Universe A, is it possible for a person to be fully morally responsible for their actions? (YES/NO)

In this case, only 14% of participants answered that it was possible for an agent to be fully morally responsible for their action in Universe A. This is strikingly different from the results obtained for the concrete case (and from the results obtained by Nahmias and colleagues). What is happening?

Nichols and Knobe’s own take on their results was that people are in fact “natural incompatibilists” but that their application of concepts such as free will and moral responsibility is biased by the affective reactions elicited by concrete cases (Nichols & Knobe, 2007). However, later research suggested that emotions elicited by concrete cases only account for a very small part of variance in participants’ attributions of free will and moral responsibility (Feltz & Cova, 2014), and that people with emotional deficits also respond differently to the abstract and concrete cases (Cova et al., 2012). A more promising explanation is thus the ‘Bypassing hypothesis’ put forward by Murray and Nahmias (2014).

Murray and Nahmias (2014) distinguish between “determinism” (as we defined it at the beginning of this paper) and “bypassing”. “Bypassing” is the claim that whatever people desire or believe is causally irrelevant to what they end up doing: even if their mental states had been completely different, the world would have caused them to act in the same way. Clearly, bypassing is incompatible with free will and moral responsibility, as it prevents us from being the source of our actions. Fortunately, determinism does not entail bypassing. Still, it might be (i) that a certain portion of participants interpret the experimental vignettes used by experimental philosophers to describe bypassing rather than determinism, and (ii) that this confusion is more frequent in the case of abstract vignettes, compared to concrete vignettes.

To put this hypothesis to test, Murray and Nichols designed a measure of bypassing (composed of items such as “In Universe A, a person’s decisions have no effect on what they end up doing” or “What Bill wants has no effect on what he ends up doing”). As predicted, they found (i) that a certain proportion of participants interpreted the vignettes as describing bypassing, and (ii) that this confusion was more frequent in the vignettes used by Nichols and Knobe than in those used by Nahmias and colleagues.^1^ Later studies showed that the difference between abstract and concrete cases was also explained (at least in part) by the fact that participants were more likely to confuse determinism with bypassing in the abstract cases (Cova & Allard, forthcoming).

Thus, a first challenge is to design vignettes that describe determinism without occasioning too much confusion with bypassing: indeed, this confusion means that certain seemingly “incompatibilist” answers do not really express a commitment to incompatibilism. But this is not the only challenge: in a more recent series of papers, Nadelhoffer and colleagues have raised the possibility that seemingly compatibilist answers might not express a genuine commitment to compatibilism either (Nadelhoffer et al., 2020, in press). According to them, it might be that participants fail to comply with the content of the vignettes and still attribute to their character some ability to act in indeterministic ways. More precisely, they think that people have an indeterministic conception of human agency (that is: they take human agency to be incompatible with determinism) and that these commitments “intrude” in participants’ interpretation of the vignettes. As such, most participants would be reluctant to fully subscribe to the determinism described in the vignettes used by experimental philosophers and would still ascribe some ‘leeway’ (i.e. possibility to escape determinism) to agents.

To test for this hypothesis, Nadelhoffer and colleagues (in press) designed their own measure of “intrusion” (composed of items such as “In Universe A, what people decide to do could have been different even if everything leading up to the decision had been exactly the same” or “In Universe A, John could have decided not to have French Fries even though his decision to have them was completely caused”). They found that, both in vignettes used by Nadelhoffer and colleagues and Nichols and Knobe, a majority of participants (57% in their first study, 71% in their second study) agreed with intrusion statements. If we take into account intrusion statements to manifest the belief that agents still had the unconditional ability to act otherwise, this means that a majority of participants failed to comply with the content of vignettes describing deterministic universes.

Additionally, Nadelhoffer and colleagues also found that a majority of participants in their study failed to distinguish determinism from bypassing (that they divided in two components: epiphenomenalism and fatalism). This accumulation of confusions should make experimenters wary of the conclusion they draw from participants’ answers: if seemingly compatibilist answers do not reflect genuine compatibilist intuitions, and if seemingly incompatibilist answers do not reflect genuine incompatibilist intuitions, it seems hard to draw any conclusion about participants’ intuitions about the compatibility of free will and determinism. This is why Murray and colleagues (forthcoming) argue for what they call “experimental nihilism”, that is: the conclusion that it is impossible to study folk intuitions about determinism because most people are unable to “get” determinism.^2^

## Time loops as a potential solution to experimental nihilism

However, before resigning ourselves to the dire conclusion that is experimental nihilism, we might wonder whether there is no way of presenting determinism that might be more familiar to participants, and might help them overcome the comprehension problems pointed in the literature. In this paper, we wanted to explore the possibility that time travel and, more precisely, time loops might be a way to communicate what determinism means to people.

A time loop is a scenario in which a protagonist is sent back to the same point in time repeatedly and thus has to live the same period of time again and again. The concept has been popularized by the movie *Groundhog Day*, in which the main character is condemned to go through the same day again and again. As such, it is a concept that is part of popular culture. However, time loops are a traditional and pedagogical way to illustrate what determinism entails. William James already used time travel as a device to explain determinism to his audience in his conference “The dilemma of determinism”:

imagine that I first walk through Divinity Avenue, and then imagine that the powers governing the universe annihilate ten minutes of time with all that it contained, and set me back at the door of this hall just as I was before the choice was made. Imagine then that, everything else being the same, I now make a different choice and traverse Oxford Street. You, as passive spectators, look on and see the two alternative universes, - one of them with me walking through Divinity Avenue in it, the other with the same me walking through Oxford Street. Now, if you are determinists you believe one of these universes to have been from eternity impossible: you believe it to have been impossible because of the intrinsic irrationality or accidentality somewhere involved in it. (James, 1907: p. 155)

Alfred Mele uses a similar strategy in his book *Free: Why science hasn’t disproved free will*, aimed at a general audience:

Discussions of a conception of free will that requires deep openness for free decision-making can quickly get very technical. I’ll try to avoid technicality here. Yesterday, George’s friends invited him to join them on a karaoke outing. George doesn’t care much for karaoke, but he likes hanging out with his friends. After giving the matter some thought, he decided to accept their invitation. Now, imagine that time (and the whole universe, actually) could be rewound in something like the way you rewind a movie you are watching on your favorite media player. And imagine that after George makes his decision, time is rewound to a moment just be­fore he decided to say yes. Everything is exactly the same as it was the first time through. But this time, what happens next—what happens when the “play” button is pressed—is that George decides to reject his friends’ invitation. This is a way to picture deep open­ness and the associated conception of having been able to have decided otherwise than the way you did. If George had deep open­ness when he made his decision, then if time could be rewound again and again for just a few moments and then played forward, he would make different decisions in some of the “replays.” (Mele, 2014).

Thus, time travel and time loops can be used to describe what a deterministic world is like. However, due to their pervasiveness in pop culture, we think that they are less likely to trigger comprehension errors than the vignettes used by experimental philosophers. Let’s take the case of bypassing: it seems that most people understand that a character who travels back in time can change the behavior of other people by changing their beliefs and desires, suggesting that they do not perceive any hint of bypassing in such scenarios. They would not withdraw blame or praise for people acting again and again in the same way because they are stuck in a time loop without being aware of it. As for intrusion, it seems that many people engaged in time travel stories expect people to behave in the same way every time, unless the main protagonist modifies the initial condition in some way. In fact, it seems that most people would be surprised or puzzled if some element differed from one repetition of the loop to the other without any explanation. This suggests that people do not feel a strong compulsion to attribute indeterministic agency to characters in such stories.

To this day, experimental philosophers have not used cases involving time loops. The vignette that gets closer to this is the *Rollback* case, which we describe earlier. But the *Rollback* case is barebone and leaves a lot to the interpretation of participants. Though one might interpret it as involving time rewound, as in the cases devised by James and Mele, it also invites other interpretation. For example, having to present this case to a general audience, one of us chose to flesh it out by using the idea that the universe goes through cycles involving the destruction of an old universe (Big Crunch) and the creation of a new one (Big Bang)—think Eternal Recurrence! Another possible interpretation is that some force “creates” multiple versions of the same universe (Giraud, 2021; see also Giraud & Cova, 2023). Thus, it is not clear that, in this underspecified version, participants interpret *Rollback* as involving time being looped or rewound, and this ambiguity might explain why the *Rollback* case has been found to elicit high rates of Bypassing and Fatalism errors (see Murray et al., forthcoming).

For all these reasons, we hoped that time loop cases would allow us to investigate people’s intuitions about determinism, free will and moral responsibility without falling into the same difficulties as previous studies. We now present the results of five studies we conducted based on this hope, before discussing their results in conclusion. Studies 1 to 3 explored participants’ spontaneous understanding of time loops, to see whether participants who had deterministic expectations about agents’ behavior in time loops would still attribute free will and moral responsibility to those agents, while Studies 4 and 5 provided them with more explicit descriptions of deterministic time loops and the causal processes underlying them.

## Open science statement

Materials, data and analysis scripts for all studies are publicly available at https://osf.io/hm7ze/. Studies conducted on Prolific were approved by University of Geneva’s ethical committee for research ethics (CUREG).

## Study 1

### Recruitment

Participants were French-speaking participants recruited through announcements on social networks (Twitter, YouTube). Calls for participation were broadcasted by Mr. Phi, a popular French YouTuber specialized in videos about philosophy (https://www.youtube.com/@MonsieurPhi). More details can be found in Giraud and Cova (2023).

### Materials and methods

The study took the form of an online survey. The original survey was in French, so we present here an English translation of the original materials (the original French survey can be found on OSF). Participants were first presented with the following question, which probed their intuitions about how humans would behave in a time loop:

Have you seen "Groundhog Day" or "Edge of Tomorrow"? These movies feature one-day time loops: the main character is aware of living the same day over and over again, while the others around him are unaware of this loop. The games "Majora's Mask" or more recently "Outer Wilds" also use this process.

Suppose that, following a physics experiment that went wrong, you have triggered a time loop and you are the only person to be aware of it... At the beginning of each loop, everything returns to exactly the same state (except the internal state of your brain which keeps the memory of the previous loops).

Do you think that all the people around you in the time loop will behave in the same way as long as you do not intervene?

- Yes: they will always repeat the same behaviors.
- No: some behaviors will be different from one time to the next.
- I don't know.

Then, participants were presented with the description of a deterministic time loop:

From one repeated day to the next, you notice that the people around you always behave in exactly the same way as long as you don’t intervene (e.g. Alice always orders the same pizza using exactly the same words).

On this particular day, Bob and Charlie are offered a sum of money to murder their respective neighbors. Bob and Charlie are not forced to do it in any way: they do not need the money badly and are not pressured or threatened. If you don't intervene, Bob and Charlie, after careful consideration, will each agree to shoot their neighbor.

You have seen this in many, many repetitions of the time loop: there are no exceptions, Bob and Charlie always behave this way in the absence of your intervention.

However, every time you have tried to dissuade Bob (by appealing to his humanity, for example), he changes his mind: he agrees with your reasons, finally refuses the proposal and does not shoot his neighbor.

On the other hand, every time you have tried to dissuade Charlie (by appealing to his humanity, for example, or for other reasons), you have never succeeded in making him change his mind. (The only time you were able to stop Charlie from shooting his neighbor was by physically restraining him or threatening him).

You have finally figured out how to get out of the time loop (and you absolutely must: the Earth will be destroyed if you don't get out!) but the procedure requires that you stay in isolation in your lab all day with no outside interaction. You follow this procedure and time is set back on its normal course. During this day, as with all the times you have not intervened, Bob and Charlie accept the proposals made to them and each goes to shoot their neighbor.

At the end of this vignette, participants were asked to rate their agreement with the four following statements (on a scale from − 2 = “Completely disagree” to 2 = “Completely agree”):

- Bob acted FREELY by shooting his neighbor. (Remember: Bob changed his mind every time you tried to talk him out of it.)
- Bob is MORALLY RESPONSIBLE for his action.
- Charlie acted FREELY by shooting his neighbor. (Remember: Charlie never changed his mind when you tried to talk him out of it.)
- Charlie is MORALLY RESPONSIBLE for his action.

After that, participants were asked to consider a case of indeterministic time loop:

Let's go back to the beginning and assume this time that you notice that the behavior of other people in the time loop is partially random. For example, Alice does not always order the same pizza (in the absence of your intervention). By taking readings, you see that she orders a Margherita in 50% of the loops; Queen 25%; Four Cheese 20%; and Hawaiian 5%.

You have seen this over a very large number of repetitions of the time loop that allowed you to establish these statistics.

You have finally discovered how to get out of the time loop. You set time back on its normal course. And you later learn that during the course of that day, Harry accepted the offer he was given and went to shoot his neighbor.

Using the statistical records you made of people's behaviors in previous loops, you find that the behaviors during that day follow the same pattern.

At the end of this vignette, participants were asked to rate their agreement with the twp following statements (on a scale from − 2 = “Completely disagree” to 2 = “Completely agree”):

- Harry acted FREELY by shooting his neighbor. (Remember: Harry had a 50% chance of deciding to shoot his neighbor and a 50% chance of deciding not to.)
- Harry is MORALLY RESPONSIBLE for his action

After completing the survey, participants were asked (but not required) to provide information about their gender, age, country of residence, education level, area of competence (Philosophy, Social sciences, Natural sciences, Formal sciences, Technology & Applied Sciences, Law, and/or Arts and Humanities), political orientation, and religiosity. They were also asked two questions assessing trait extraversion (drawn from Gosling et al., 2003), and two questions about their opinion regarding the Newcomb’s paradox (that the youtuber MrPhi had presented in a recent video).

### Results

In total, 2,567 participants completed the survey and accepted that their data be used for research purposes (2071 men, 427 women, 43 ‘other’, and 26 unidentified; *M*_age_ = 28.84, *SD*_age_ = 9.44). 84.0% declared France as their country of residence, 5.1% Belgium, 3.0% Switzerland, and 1.8% Canada. Only 7.1% declared Philosophy as one of their areas of competence.

To the first question, 73.1% answered that people in the time loop would *always* repeat the same behavior, 17.5% answered that some behaviors would differ from one loop to the next, and 9.5% answered that they did not know. Thus, overall, participants had no trouble thinking that human behavior would repeat themselves identically within a time loop, suggesting that beliefs about indeterministic agency did not “intrude” too much in participants’ understanding of what a time loop is.

We then looked at participants’ judgments about the free will and moral responsibility of agents in time loops. Results are presented in Table 1. As one can see, people tended to attribute both free will and moral responsibility to agents in deterministic time loops, irrespective of their propensity to change their minds, and did not attribute more free will and moral responsibility to the agent in an indeterministic time loop. Moreover, participants’ answer to the first question (whether they considered that agents in a time loop would always act the same way) did not significantly impact their free will and moral responsibility judgments.

**Table 1:** Mean and standard deviations for the free will and moral responsibility questions (Study 1)

	Bob (Deterministic, can change his mind)	Charlie (Deterministic, will not change his mind)	Harry (Indeterministic)
Free will	1.15 (1.19) 80.1%	1.24 (1.16) 82.6%	1.20 (1.11) 80.4%
*Difference (FW)	Δ = − 0.05	Δ = − 0.04	Δ = − 0.08
Moral responsibility	1.54 (0.84) 91.7%	1.59 (0.81) 92.8%	1.54 (0.82) 91.3%
*Difference (MR)	Δ = + 0.07	Δ = + 0.02	Δ = − 0.03

Percentages indicate the % of participants who gave an answer superior to the midpoint (= 0). The “Difference” rows indicate for each score the difference in means between participants who answered that people will always act the same way in a time loop and others. None of these differences was statistically significant

### Discussion

Overall, the results of Study 1 suggest two main results. The first is that most participants seem to suppose that people in a time loop will always act in the same way—and thus that, in absence of any interference, the same initial conditions will yield the same results, even in human affairs. This suggests that people might not have a deeply ingrained indeterministic view of human agency or, at least, that this view does not interfere with their understanding of cases involving time loops. The second is that, even when we specified that the time loop was deterministic and that the same initial conditions would always yield the same results, most participants attributed free will and moral responsibility to agents. Moreover, participants did not attribute more free will and moral responsibility when we specified that the time loop was indeterministic. This suggests that most people do not see determinism as relevant to free will and moral responsibility.

Now, there are limitations to our study. The first is the population: people who answered our survey were people with a particular interest in philosophy, and this might draw their intuitions away from commonsense intuitions. The second is the method: we did not describe what a time loop is, but rather directed participants at well-known works of fiction (such as *Groundhog Day*) as an example. Given that these works of fiction are all based on the premise that events in a time loop will repeat themselves identically in absence of outside interference, this might have prompted participants to endorse a deterministic view of time loop that does not really reflect their intuitive view of human agency. However, this second worry only sheds doubt on participants’ answers to the first question: it leaves intact the fact that, even when prompted to think about time loops in a deterministic way, most of our participants still attributed free will and moral responsibility to agents.

We tried to correct both shortcomings in Study 2.

## Study 2

### Materials and methods

The study took the form of an online survey. Participants were first introduced to the story of John, a physicist stuck in a time loop:

Imagine that John is a physicist. After an experiment that went terribly wrong, John has been stuck in a time loop. He has been condemned to live the same day again and again (let’s say January 15th, 2022). Every time John reaches midnight, he’s sent 24 hours back in the past (even if he died in the meantime). And every time the state of the world at midnight is identical to what it was the first time John started the day, except for John’s mind and brain, which allows him to keep his memory from the previous times he went through the day.

Imagine that John is going through his first repetition of the loop. He remembers that, the first time he went through the day, the postman rang at his neighbor Bob's door at 10:30 am.

John is wondering whether, if he does nothing to interfere, the postman will ring at his neighbor Bob's door every time John goes through the loop.

Just after, participants were asked the following question:

Imagine that John goes through 100 additional loops (days) while staying at home and doing NOTHING to intervene in other people's lives.

On how many of these 100 loops will the postman ring at Bob the neighbor's door at 10:30 am?

- None of them (0 loops)
- Between 1 and 25 loops
- Between 26 and 50 loops
- Between 51 and 75 loops
- Between 76 and 99 loops
- All of them (100 loops)

Participants were asked to justify their answer. Then, they were randomly assigned to one of two conditions (Confident Al / Hesitant Hal). The two conditions are presented in Table 2.

**Table 2:** Experimental conditions for Study 2

Experimental conditions for study 2	
Confident Al	Hesitant Al
Now, imagine that John is going through his first repetition of the loop. He remembers that, the first time he went through the day, someone killed Bob by shooting him in the head at 2 pm John does not know who killed Bob. In fact, the killer is Al, another neighbor. Al was actually paid by Bob’s heir to kill Bob so that his heir could receive his legacy. Al gladly accepted because not only did he need the money to pay his rent, he also hated Bob for making too much noise and not sharing the same political opinions John is wondering whether, if he does nothing to interfere, the killer will shoot Bob in the head every time	Now, imagine that John is going through his first repetition of the loop. He remembers that, the first time he went through the day, someone killed Bob by shooting him in the head at 2 pm John does not know who killed Bob. In fact, the killer is Al, another neighbor. Al was actually paid by Bob’s heir to kill Bob so that his heir could receive his legacy. Al accepted after a lot of hesitation because he actually liked Bob a lot and was friend with him. However, Al really needed the money to pay his rent and not become homeless. Until the last minute, he hesitated and did not know whether he would be able to do it John is wondering whether, if he does nothing to interfere, the killer will shoot Bob in the head every time

In both conditions, participants were asked:

Imagine that John goes through 100 additional loops (days) while staying at home and doing NOTHING to intervene in other people's lives. On how many of these 100 loops will Al shoot Bob in the head at 2pm?

- None of them (0 loops)
- Between 1 and 25 loops
- Between 26 and 50 loops
- Between 51 and 75 loops
- Between 76 and 99 loops
- All of them (100 loops)

Then, participants were presented with the following text:

Imagine that John is going through his 200th loop. As he is working on a way to break the loop and to stop the day from repeating itself, he spends the whole day at home, and does NOT interfere in other people's lives.

At 2pm, Al shoots Bob in the head.

Just after, they were asked to answer five questions about moral responsibility, blame, punishment, free will, and the ability to do otherwise:

- *Moral Responsibility:* To which extent is Al morally responsible for shooting Bob in the head? (from 0 = “Not responsible at all” to 6 = “Completely morally responsible”) (they were asked to justify their answer)
- *Blame:* To which extent does Al deserve blame for shooting Bob in the head? (from 0 = “Not at all” to 6 = “A lot”)
- *Punishment:* To which extent does Al deserve to be punished for shooting Bob in the head? (from 0 = “Not at all” to 6 = “A lot”)
- *Free Will:* To which extent do you agree with the following statement: “Al killed Bob of his own free will.” (from − 3 = “Strongly disagree” to 3 = “Strongly agree”)
- *Could have done otherwise:* To which extent do you agree with the following statement: “Albert could have chosen not to kill Bob.” (from − 3 = “Strongly disagree” to 3 = “Strongly agree”)

Then, participants were asked two questions that served as attention checks (“What is the name of the character locked in a time loop?”, “What is John's job?”).

At the end of the study, participants were asked to fill various scales presented in random order: the “Satisfaction with life” scale (Diener et al., 1985), the “Presence of Meaning” subscale from the “Meaning-in-Life Questionnaire” (Steger et al., 2006), a measure of performance in the workplace drawn from Stillman et al. (2010), the “Generalized self-efficacy scale” (Schwarzer & Jerusalem, 1995), a measure of desire to inflict cruel punishment to criminal offenders drawn from Clark et al. (2017), three subscales about the treatment of criminal offenders (Gerber & Jackson, 2013), and The “Free Will” subscale and “Determinism” subscale of the “Free Will Inventory” (Nadelhoffer et al., 2014). We also added an additional item, supposed to measure participants’ endorsement of the Principle of Sufficient Reason. Participants were also asked whether they currently were in a romantic relationship and, if yes, were asked to fill the 4-item version of the “Couples Satisfaction Index” (Funk & Rogge, 2007). All these data were collected for another research project and won’t be discussed here.

Finally, participants were asked to indicate their age and gender.

### Results

246 participants recruited through Prolific Academic (US and UK residents only) completed our survey. After excluding participants who failed at least one of four attention checks, we were left with 218 participants (105 men, 110 women, 3 others; *M*_age_ = 38.19, *SD*_age_ = 13.07).

For the first question (“On how many of these 100 loops will the postman ring at Bob the neighbor's door at 10:30 am?”), 200 participants (91.7%) answered that this would occur on 100 loops out of 100.

For the second question (“On how many of these 100 loops will Al shoot Bob in the head at 2 pm?”), 99 participants out of 106 (93.4%) answered that Al would always shoot Bob in the CERTAIN condition, against 98 out of 112 (87.5%) in the HESITANT condition.

Keeping only the participants who answered that Al would always shoot Bob in the head, we looked at these participants’ answers to the five questions about free will and moral responsibility. Results are presented in Table 3.

**Table 3:** Mean and standard deviations for the moral responsibility, blame, punishment, free will, and choice questions, in function of experimental conditions (Confident Al vs. Hesitant Al)

	Confident Al	Hesitant Al
Moral responsibility	5.05 (1.75) 84.7%	4.67 (2.16) 76.8%
Blame	5.71 (0.77) 96.9%	5.58 (1.17) 91.9%
Punishment	5.71 (0.64) 98.0%	5.67 (1.14) 94.9%
Free will	2.43 (1.11) 94.9%	2.48 (1.15) 96.0%
Could have chosen	2.64 (1.05) 93.9%	2.60 (1.08) 93.9%
*N*	98	99

% indicates the percentage of participants who gave an answer superior to the midpoint

### Discussion

As in Study 1, most participants answered that the same action (e.g. Al shooting Bob in the head) would repeat themselves in all loops. And, as in Study 1, most participants still attributed free will and moral responsibility to agents in these deterministic loops. Again, this suggests that people are not deeply committed to an indeterministic view of human agency (or, in any case, that this view does not “intrude” in their understanding of our vignette), and that they do not hesitate to attribute free will and moral responsibility to agents whose actions are determined by the past and initial conditions.

However, one limitation of our study is the duration of the time loop (one day). On certain libertarian conceptions of human agency (e.g. Kane, 1998), most of our daily actions are determined by our mental states and character—however, our character is the product of past indeterministic decisions that have contributed to shape them. Thus, we could imagine that Al’s character determines him to kill Bob, but that Al still *freely* kills Bob in a libertarian way because Al’s character has been shaped by indeterministic decisions that Al made before the time loop started (for example, when he was young).

It might be possible that some people endorse such a conception of human agency. To test for this possibility, we designed a new study in which the time loop lasted 30 years and began before the birth of the agent (i.e. Al).

## Study 3

### Materials and methods

The study took the form of an online survey. As in Study 2, participants were introduced to the case of John, a physicist stuck into a time loop—except this time the time loop lasted 30 years and John had no way of interfering with his environment:

Imagine that John is a physicist. He has been sent in space 35 years ago to conduct secret physics experiments in a space station orbiting earth. Unfortunately, due to a collision with an asteroid, the space station has stopped working properly and has been propelled in the depths of space. John has lost all ways to send messages to Earth and no one knows where he is. However, he is still able to receive news from Earth. Thus, he can know what happens on Earth but cannot intervene in any way in what happens on Earth. He has been living since then, alone in the lost space station.

However, John has been continuing his physics experiments. Unfortunately, after an experiment that went terribly wrong, John has been stuck in a time loop. Every time John reaches January 15th, 2022, he's sent 30 years back in the past (even if he died in the meantime), on January 15th, 1992. And every time the state of the world on January 15th, 1992 is identical to what it was the first time John started experiencing it, except for John's mind and brain, which allows him to keep his memory from the previous times he went through these 30 years.

Imagine that John is going through his first repetition of the loop. He (correctly) remembers that, the first time he went through this 30 years period, he learned from the radio that on March 1st, 2019, Brett, a 19-years old man from another country, succeeded in solving a complex math theorem.

John is wondering whether Brett will solve the complex math theorem every time John goes through the loop.

Participants were then asked their first question:

Imagine that John goes through 100 additional loops (30-years periods) while still being UNABLE to intervene in other people’s lives. (Because he's stuck on his space station without any means of contacting Earth.) On how many of these 100 loops will the radio report about Brett solving the complex math theorem on March 1st, 2019?

- None of them (0 loops)
- Between 1 and 25 loops
- Between 26 and 50 loops
- Between 51 and 75 loops
- Between 76 and 99 loops
- All of them (100 loops)

Participants were asked to justify their answer. Then, they were randomly assigned to one of two conditions (Confident Al / Hesitant Al). The two conditions are presented in Table 4.

**Table 4:** Experimental conditions for study 3

Experimental conditions for Study 3	
Confident Al	Hesitant Al
Now, imagine that John is going through his first repetition of the loop. He remembers that, the first time he went through this 30-years period, he heard on the radio that a 21-years old man named Al Smith killed his neighbor Bob on July 1st, 2021 Al was actually paid by Bob’s heir to kill Bob so that his heir could receive his legacy. Al gladly accepted because not only did he need the money to pay his rent, he also hated Bob for making too much noise and not sharing the same political opinions John is wondering whether, if he does nothing to interfere, the killer will shoot Bob in the head every time	Now, imagine that John is going through his first repetition of the loop. He remembers that, the first time he went through this 30-years period, he heard on the radio that a 21-years old man named Al Smith killed his neighbor Bob on July 1st, 2021 Al was actually paid by Bob’s heir to kill Bob so that his heir could receive his legacy. Al accepted after a lot of hesitation because he actually liked Bob a lot and was friend with him. However, Al really needed the money to pay his rent and not become homeless. Until the last minute, he hesitated and did not know whether he would be able to do it John is wondering whether, if he does nothing to interfere, the killer will shoot Bob in the head every time

In both conditions, participants were asked the following question:

Imagine that John goes through 100 additional loops while being stuck in his space station and doing NOTHING to intervene in other people's lives. On how many of these 100 loops will Al shoot Bob in the head on July 1st, 2021?

- None of them (0 loops)
- Between 1 and 25 loops
- Between 26 and 50 loops
- Between 51 and 75 loops
- Between 76 and 99 loops
- All of them (100 loops)

Participants were then presented with the following text:

Imagine that John is going through his 200th loop. As he is working on a way to break the loop and to stop the 30-years period from repeating itself, he spends the whole time stuck in his space station, and does NOT interfere in other people's lives.

On July 1st, Al shoots Bob in the head.

Participants were asked the same questions as in Study 2 (about Al’s moral responsibility, blame, punishment, and free will), except that the free will question was now asked on a scale from 0 = “Not at all” to 6 = “A lot”) and that we forgot to include the question about the ability to choose otherwise. Participants were asked to justify their answer to the moral responsibility question.

Then, participants were asked two questions that served as attention checks (“What is the name of the character locked in a time loop?”, “What is John’s job?”).

At the end of the survey, participants were asked to fill the same scales as in Study 2.

### Results

248 participants recruited through Prolific Academic (US and UK residents only) completed our survey. After excluding participants who failed at least one of four attention checks, we were left with 229 participants (110 men, 116 women, 3 others; *M*_age_ = 36.16, *SD*_age_ = 12.36).

For the first question (“On how many of these 100 loops will the radio report about Brett solving the complex math theorem on March 1st, 2019?”), 192 participants (83.8%) answered that this would occur on 100 loops out of 100.

For the second question (“On how many of these 100 loops will Al shoot Bob in the head at 2 pm?”), 93 participants out of 114 (81.6%) answered that Al would always shoot Bob in the CERTAIN condition, against 88 out of 115 (76.5%) in the HESITANT condition.

Keeping only the participants who answered that Al would always shoot Bob in the head, we looked at these participants’ answers to the four questions about moral responsibility, blame, punishment, and free will. Results are presented in Table 5.

**Table 5:** Mean and standard deviations for the moral responsibility, blame, punishment and free will questions, in function of experimental conditions (Confident Al vs. Hesitant Al)

	Confident Al	Hesitant Al
Moral responsibility	4.83 (2.23) 71.0%	4.16 (2.57) 78.4%
Blame	5.60 (1.23) 92.5%	5.48 (1.23) 96.6%
Punishment	5.56 (1.24) 94.4%	5.49 (1.23) 96.6%
Free Will	5.47 (1.26) 97.8%	5.35 (1.27) 98.9%
*N*	93	88

% indicate the percentage of participants who gave an answer superior to the midpoint

### Coding

To check whether participants understood the case and questions they were presented with, the second author on this paper was tasked with coding participants’ justifications to the Moral Responsibility question. Methods and results for this analysis are presented in Supplementary Materials.

### Discussion

Using a 30-year rather than a one-day time loop did not change most of our conclusions: most participants expected the same actions to repeat themselves in all 100 loops, including murder. Moreover, most of those participants attributed free will and moral responsibility (as well as blame and punishment) to agents. If we take people answering that the loop will always repeat itself identically to accept the idea that our scenario describes a deterministic universe, then this suggests that most of our participants did not see determinism as a threat to free will and moral responsibility.

However, one criticism that can be made against Studies 2 and 3 is that we did not tell participants that the universe was deterministic: we simply selected participants who answered that the loop would repeat itself every time. It might be that these participants differ from others by being already more likely to see free will and moral responsibility as compatible with determinism. Thus, in Study 4, we keep the 30-year loop case but try to make clear to participants that the main character is living in a deterministic universe.

Also, in Studies 1 to 3, we did not directly assess whether participants made comprehension mistakes, such as confusing determinism and bypassing. Thus, we added measures of bypassing, fatalism and intrusion in Study 4.

## Study 4

### Materials and methods

The study took the form of an online survey. The study began exactly as Study 3: participants were introduced to the case of John, the physicist stuck in a 30-year time loop. They were then asked the same question about Brett solving the difficult math theorem:

Imagine that John goes through 100 additional loops (30-years periods) while still being UNABLE to intervene in other people's lives. (Because he’s stuck on his space station without any means of contacting Earth.) On how many of these 100 loops will the radio report about Brett solving the complex math theorem on March 1st, 2019?

- None of them (0 loops)
- Between 1 and 25 loops
- Between 26 and 50 loops
- Between 51 and 75 loops
- Between 76 and 99 loops
- All of them (100 loops)

Once they answered this question, participants were presented with a text explaining that Brett’s behavior would be identical in all loops, because John’s universe is a universe in which the same initial conditions inevitably lead to the same consequences, even when these consequences involve human action:

Going through 100 repetitions of the loop, John has observed that Brett solved the complex math theorem on March 1st, 2019 in 100 loops out of 100. From these observations (as well as numerous other repetitions), he (rightly) concludes that, as long as he does not interfere, the exact same things will happen again in each time loop.

This is because the universe John lives in is such that the same causes will always bring the same consequences. Thus, since each loop brings the universe back to the very same state it was on January 15th, 1992, the same things have to happen again, since the same initial conditions will lead to the same results.

This is not to say that things are fated to happen: if John could interfere with what happens on Earth, he could modify the course of things. For example, he could kill Brett before Brett solves the theorem (or simply convince him not to learn mathematics). Changing the initial conditions would change what happens next.

However, since John is locked in his space station, he cannot interfere with what happens on Earth, and thus the same initial condition will bring the same consequences in each time loop. Even human actions will be the same every time, because, in John's universe, the way humans act and the decisions they make depend on what happened before.

Participants were then asked the Similarity question:

To which extent do you think our own universe is like John's universe?

- Our universe is just like John's universe: what happens depend on what happened before and the same causes will lead to the same consequences.
- Our universe is different from John's universe: sometimes, the same causes might lead to different consequences.
- I don't know.

After that, to check that participants understood that, in John’s universe, the same events would repeat themselves in all loops, we asked them the same question about Bob’s murder as in Studies 2 and 3:

Now, imagine that John is going through his first repetition of the loop. He remembers that, the first time he went through this 30-years period, he heard on the radio that a 21-years old man named Albert Smith killed his neighbor Bob on July 1st, 2021.

Albert was actually paid by Bob’s heir to kill Bob so that his heir could receive his legacy. Albert accepted and shot Bob.

John is wondering whether, if he does nothing to interfere, Albert will shoot Bob in the head every time.

Imagine that John goes through 100 additional loops while being stuck in his space station and doing NOTHING to intervene in other people's lives. On how many of these 100 loops will Albert shoot Bob in the head on July 1st, 2021?

- None of them (0 loops)
- Between 1 and 25 loops
- Between 26 and 50 loops
- Between 51 and 75 loops
- Between 76 and 99 loops
- All of them (100 loops)

Then, participants were presented with the description of the Albert shooting Bob and asked the same questions as in Study 3, about moral responsibility, blame, punishment and free will (the name “Al” was replaced by “Albert” because analysis of qualitative data in Study 3 suggested a few participants took “Al" to refer to an artificial intelligence). We also asked people whether they agreed with the statement “Albert could have chosen not to kill Bob" (YES/NO) and to justify their answer. Participants who answered ‘YES’ were then presented with the following question:

When you answered that "Albert could have chosen not to kill Bob", what exactly did you mean?

- That, if (and only if) Albert's desires, beliefs and/or character had been different, it might have happened that Albert chose not to kill Bob.
- That it might have happened that Albert chose not to kill Bob even if Albert's desires, beliefs and character had been exactly the same.
- Something else.

The first answer was supposed to capture the “conditional” interpretation of could have done otherwise. The second was supposed to capture the “unconditional” interpretation.

Finally, participants were asked to rate their agreement (on a scale from − 3 = “Strongly disagree” to 3 = “Strongly agree”) with 12 statements adapted from Nadelhoffer and colleagues (in press). 4 statements measured confusion with bypassing, 4 measured confusion with fatalism, and 4 measured intrusion. The intrusion items were the following:

- Albert could have decided not to kill Bob even if everything (including the laws of nature) had been exactly the same prior to his decision.
- There was at least a slight chance that Albert could have chosen not to kill Bob even if everything (including the laws of nature) had been exactly the same prior to his decision.
- It was open for Albert to choose not to kill Bob at the exact moment he decided to kill him.
- Albert could have decided not to kill Bob even though his decision to kill him was caused by what happened before.

At the end of the study, participants were asked two questions that served as attention checks.

### Results

261 participants recruited through Prolific Academic (US and UK residents only) completed our survey. After excluding participants who failed at least one of two attention checks, we were left with 244 participants (119 women, 123 men, 2 others; *M*_age_ = 40.63, *SD*_age_ = 13.61).

For the first question (“On how many of these 100 loops will the radio report about Brett solving the complex math theorem on March 1st, 2019?”), 198 participants (81.1%) answered that this would occur on 100 loops out of 100.

We then excluded participants based on their answer to the question about the number of loops in which Albert would kill Bob, to only keep those who answered that Albert would kill Bob in 100 loops out of 100 (since it was explained that, in John’s world, the same causes always bring the same consequences). We were left with 216 participants, as 28 (11.5%) failed this comprehension test.

Of these 216, 103 (47.7%) answered that our universe was different from John’s, 92 (42.6%) answered that our universe was like John’s, and 21 (9.7%) answered that they did not know.

Answers to the moral responsibility, free will, blame and punishment questions are presented in Table 6. We compared the answers of participants who answered that our world was like John’s to the answers of participants who answered that it was different. Four Welch t-tests and a Chi-square test found no significant difference between the two groups.

**Table 6:** Mean and standard deviations for the moral responsibility, blame, punishment, free will and ability to have done otherwise questions, in function of participants’ answers to the similarity question (Study 4)

	All	Similar	Different
Moral responsibility	4.38 (2.56) 73.1%	4.25 (2.68) 71.7%	4.50 (2.49) 74.8%
Blame	5.33 (1.64) 89.4%	5.32 (1.70) 90.2%	5.37 (1.58) 88.3%
Punishment	5.39 (1.64) 89.4%	5.39 (1.64) 90.2%	5.42 (1.58) 88.3%
Free will	5.27 (1.45) 89.8%	5.24 (1.39) 90.2%	5.38 (1.34) 91.3%
Could have done otherwise	94.4%	90.2%	97.1%
Bypassing	− 1.54 (1.28) 8.3%	− 1.62 (1.28) 7.6%	− 1.55 (1.22) 7.8%
Fatalism	− 0.76 (1.49) 25.5%	− 0.44 (1.56) 32.6%	− 1.15 (1.37) 18.5%
Intrusion	1.18 (1.44) 78.2%	0.74 (1.57) 66.3%	1.63 (1.27) 87.4%
*N*	207	86	102

% indicates the percentage of participants who gave an answer superior to the midpoint

As can be seen in Table 6, 94.4% of the remaining participants answered that Albert could have chosen not to kill Bob. Among those participants, 55.4% chose the “conditional” interpretation of “could have done otherwise,” while 41.1% chose the “unconditional” interpretation of “could have done otherwise”, and 3.4% answered “something else”. Whether participants chose the conditional or unconditional interpretation did not significantly affect their answer to the moral responsibility, blame, punishment and free will questions (*p* > 0.23).

Finally, we examined participants’ answers to the Bypassing, Fatalism, and Intrusion questions. Results are presented in Table 6. Curiously, Fatalism scores were significantly higher (*p* = 0.001) and Intrusion scores were significantly lower (*p* < 0.001) for participants who answered that our universe is just like John’s Universe. To determine which comprehension errors drove participants’ judgments, we ran four multiple regression analyses with all three types of errors (Bypassing, Fatalism, and Intrusion) as predictors and participants’ answers to the moral responsibility, blame, punishment, and free will questions. Results are presented in Table 7. Overall, only confusion with bypassing seemed to impact participants’ judgments (except for Free Will attributions, in which Intrusion played some role).

**Table 7:** Standardized coefficients and p-values for four multiple regression analyses with Bypassing, Fatalism and Intrusion scores as predictors, and Moral responsibility, Blame, Punishment, and Free will as dependent variables (Study 4)

	Bypassing	Fatalism	Intrusion	*R*^2^
Moral responsibility	β = − 0.29 *p* < .001***	β = 0.04 *p* = .61	β = 0.01 *p* = .84	0.07
Blame	β = − 0.36 *p* < .001***	β = -0.03 *p* = .70	β = 0.05 *p* = .47	0.15
Punishment	β = − 0.40 *p* < .001***	β = 0.03 *p* = .68	β = 0.10 *p* = .15	0.16
Free will	β = − 0.36 *p* < .001***	β = 0.06 *p* = .49	β = 0.15 *p* = .03*	0.15

## Study 5

The results of Study 4 suggested that, even when directly asked to imagine a world in which time loops would always produce the same events and presented with a deterministic explanation for this fact, most participants still answered that agents in time loops acted freely and were morally responsible for their actions. This suggests that people see no contradiction between determinism and moral responsibility.

But did the use of time loops successfully prevent some of the comprehension errors pointed out by Nadelhoffer and colleagues? Bypassing and Fatalism scores were low, but Intrusion scores were still high. One possibility is that time loops are not proper ways of describing determinism, and lead participants to attribute indeterministic agency to agents (even though they agree that the agents would act the same in every replay of the time loop). Another is that Intrusion scores do not measure what they were intended to measure (i.e. the belief that agents have the unconditional ability to do otherwise), but also captures beliefs that are perfectly compatible with determinism (i.e. the belief that agents have the conditional ability to do otherwise). This is at least what participants’ answers to our questions about the ability to do otherwise suggest: they claim that the agent could have done otherwise, but most of them only claim it in the sense that, had the agents’ mental states been different, he might have acted otherwise. One way to test whether Intrusion measures really measure what they intend to measure (and whether our vignettes are successful in preventing comprehension errors) is to compare them to another related measure. In Study 5, we used a modified version of the Determinism subscale of the Free Will Inventory (Nadelhoffer et al., 2014) to assess participants’ perception of determinism (and the unconditional ability to do otherwise).

We also used Study 5 as an opportunity to try a variation on the case used from Studies 2 to 4. To get closer to the kind of situations described by James and Mele in introduction, we designed a *Rewind* case, in which time is described as “rewound” rather than “looping”, and in which nobody escapes the rewind.^3^ We also focused on minor moral violations, rather than serious crimes, as it has sometimes been suggested in the literature that people might be more driven to compatibilist intuitions when faced with serious moral violations compared to minor moral violations (Nichols & Knobe, 2007), even though subsequent replication studies have shown that this effect is at best very small (Feltz & Cova, 2014).

### Materials and methods

The study took the form of an online survey. Participants were presented with the following story:

Imagine that John is a physicist who is convinced that time travel is possible. He has been working on a time machine for several years now.

On **May 1st, 2023**, John wakes up at 8am. He then walks to the nearby university, where his lab is located. While he is on his way, *at 8:30am precisely*, it begins raining.

When he arrives at his office, John puts his lunch (a sandwich and a slice of cheesecake) in his personal fridge. Then, he goes to his lab to work on his machine. At *11:30am*, while John is in his lab, Fred, a colleague of John who feels hungry, enters John's office and steals John's slice of cheesecake. When John goes back to his lab to have lunch at 1:00pm, he discovers with disappointment that the slice of cheesecake has gone.

John then goes back to his lab to work on his machine. *At 4:00pm*, he hears a very loud noise and opens his lab's window to look for the source of the sound. It turns out that it was a stray dog who had knocked over a large garbage can.

*At 6:00pm*, someone knocks at John's door. It turns out it's Arthur, another of John's colleagues who is worried about John, as John spends most of his time working. He has brought John a cup of coffee and a cupcake to cheer him up and to force him to take a break. John accepts gratefully.

Despite all that, John continues to work on his machine, having the feeling that he is nearing his goal. At 8:00pm, he connects two cables and presses his machine's POWER button. At this very moment, something unexpected happens: the machine works, but not in the way John intended. It actually *rewinds* time: time is rewound 12 hours back, so that **everything in the universe goes back to the exact same state it was at 8am this very morning**. This includes the brain of John and every other living being in the universe: no one has any recollection of what happened in the last 12 hours. Everything, every single atom and molecule in the universe is back at the place and state it was 12 hours ago, at 8:00 am (and, of course, all functional clocks in John's vicinity indicates that it is 8:00 am).

Thus, it is 8:00am again and, having no recollection of what happened (since his mind is back at the state it was 12 hours ago), John wakes up to go to work.

After reading the story, participants were asked the following question about four events: the rain starting to fall at 8.30am, Fred stealing the slice of cheesecake at 11:30am, the dog knocking over the large garbage can at 4:00 pm, and Arthur offering John a cupcake at 6:00 pm:

The first time John went through this day, Fred stole his slice of cheesecake at 11:30am. What are the chances that, in this second run of May 1st, 2023, Fred will steal the slice of cheesecake at 11:30am?

- Fred will necessarily steal the slice of cheesecake at 11:30am.
- Fred will probably steal the slice of cheesecake at 11:30 am, but there is a chance he might not steal it this time.
- There is a 50% chance that Fred will steal the slice of cheesecake at 11:30am, and a 50% chance that he won't.
- It is unlikely that Fred will steal the slice of cheesecake at 11:30 am, but there is still a chance he might steal it.
- It is absolutely impossible that Fred will steal the slice of cheesecake at 11:30am.

Then, participants were given the following description of John’s universe:

It turns out that, on this second run of May 1st, 2023, everything happened exactly in the same way as in the first run: it started raining at 8:30am, Fred stole the slice of cheesecake at 11:30am, the dog knocked over the large garbage can at 4pm, and Arthur offered John a cup of coffee and cupcake at 6pm. At 8pm, John powered his machine on, and (once again) time was rewinded and everything was brought back to the state it was 12 hours before.

Thus, May 1st, 2023 was replayed a third time, a fourth time, a fifth time, and so on. And in every replay, the same things happened over and over again, without any change: on every replay, it started raining at 8:30am, Fred stole the slice of cheesecake at 11:30am, the dog knocked over the large garbage can at 4pm, Arthur offered John a cup of coffee and cupcake at 6pm, and John powered on his machine at 8:00pm, rewinding everything back at the state it was 12 hours ago.

This is because the universe John lives in is such that the same causes will always bring the same consequences. In this universe, the same conditions and the same laws of nature produce the exact same outcomes. Thus, since John's machine rewinds the universe back to very same state it was on May 1st, 2023 at 8am, the same things happen again and again, since the same initial conditions lead to the same results.

As in Study 4, participants were asked to which extent John’s universe is similar to ours. Then, they were asked to comprehension questions, one about Fred stealing the slice of cheesecake, and one about Arthur offering John a cupcake:

Imagine that John goes through the day 100 more times. On how many of these 100 replays will [Fred steal his slice of cheesecake at 11:30am / Arthur offer him a cupcake at 6:00pm]?

- None of them (0 replays)
- Between 1 and 25 replays
- Between 26 and 50 replays
- Between 51 and 75 replays
- Between 76 and 99 replays
- All of them (100 replays)

Then, participants were told John went through his 200^th^ replay of the day and that everything happened exactly in the same way it happened the first time. They were asked to which extent Fred was morally responsible for stealing the cheesecake (on a scale from 0 = “Not morally responsible at all” to 6 = “Completely morally responsible”), whether Fred deserved blame for stealing the cheesecake (on a scale from − 3 = “Strongly disagree” to 3 = “Strongly agree”), and whether Fred stole the cheesecake of his own free will (on a scale from − 3 = “Strongly disagree” to 3 = “Strongly agree”). Similarly, they were asked to which extent Arthur was morally responsible for having offered John a cupcake (on a scale from 0 = “Not morally responsible at all” to 6 = “Completely morally responsible”), whether Arthur deserved praise for offering the cupcake (on a scale from − 3 = “Strongly disagree” to 3 = “Strongly agree”), and whether Arthur offered the cupcake of his own free will (on a scale from − 3 = “Strongly disagree” to 3 = “Strongly agree”).

After that, participants were asked whether they agreed with the following statement: “Fred could have chosen not to steal John's slice of cheesecake” (YES/NO). Those who answered ‘YES’ were presented with the following question:

When you answered that "Fred could have chosen not to steal John's slice of cheesecake", what exactly did you mean?

- That, if (and only if) Fred's desires, beliefs and/or character had been different, it might have happened that Fred chose not to steal the slice of cheesecake.
- That, if (and only if) what happened in the past had been different, it might have happened that Fred chose not to steal the slice of cheesecake.
- That it might have happened that Fred chose not to steal the slice of cheesecake even if Fred's desires, beliefs and character and everything else that happened in the past had been exactly the same.
- Something else.

As in Study 4, participants were asked to rate their agreement (on a scale from − 3 = “Strongly disagree” to 3 = “Strongly agree”) with 4 Bypassing, 4 Fatalism, and 4 Intrusion items. The Intrusion items were the following:

- Fred could have decided not to steal the slice of cheesecake even if everything (including the laws of nature) had been exactly the same prior to his decision.
- There was at least a slight chance that Fred could have chosen not to steal the slice of cheesecake even if everything (including the laws of nature) had been exactly the same prior to his decision.
- It was open for Fred to choose not to steal the slice of cheesecake at the exact moment he decided to steal it.
- Fred could have decided not to steal the slice of cheesecake though his decision to steal it was caused by what happened before.

We also asked participants to rate their agreement with five items drawn and modified from the Determinism subscale of the Free Will Inventory (Nadelhoffer et al., 2014). The Determinism subscale is a validated measure of belief in determinism and typically used to measure participants’ belief that *our* world is deterministic, but we modified the items to be about John’s universe (e.g. “In John’s Universe, given the way things were at the Big Bang, there is only one way for everything to happen in the universe after that”).

At the end of the study, participants were asked two questions that served as attention checks.

### Results

245 participants recruited through Prolific Academic (US residents only) completed our survey. After excluding participants who failed at least one of two attention checks, we were left with 202 participants (100 women, 101 men, 1 other; *M*_age_ = 40.84, *SD*_age_ = 14.40).

For the first batch of questions (“What are the chances that, in this second run of May 1st, 2023, [event *E* will happen] at [given time]?”), answers are presented in Table 8. As can be seen, most participants thought that all four types of events would necessarily happen again in the second run of the day.

**Table 8:** Distribution of answers for participants’ estimates of the chances that a certain event will occur again in the second run of the day (Study 5)

	Rain (%)	Fred (%)	Dog (%)	Arthur (%)
Necessarily	77.2	68.3	65.8	70.3
Probably	16.3	25.2	20.3	23.3
Fifty-fifty	4.5	4.0	7.4	4.5
Probably not	1.0	1.5	5.9	1.0
Impossible	1.0	1.0	0.5	1.0

Participants were then presented with the description of John’s universe. 52.5% answered that our universe was different from John’s, 33.2% answered that our universe was just like John’s, and 14.4% answered that they didn’t know. After that, we excluded participants who failed to answer that Fred would steal the cheesecake and Arthur would offer a cupcake in 100 replays out of 100. We were left with 179 participants, as 23 (11.4%) failed this comprehension test. Of these 179, 51.4% answered that our universe was different from John’s, 34.6% answered that our universe was like John’s, and 14.0% answered that they did not know.

Answers to the moral responsibility, blame/praise and free will questions are presented in Table 9. We compared the answers of participants who answered that our world was like John’s to the answers of participants who answered that it was different. Six Welch t-tests found no significant difference between the two groups.

**Table 9:** Mean and standard deviations for the moral responsibility, blame/praise, free will and ability to have done otherwise questions, in function of participants’ answers to the similarity question (Study 5)

	All	Similar	Different
Moral responsibility (Fred)	5.22 (1.69) 88.3%	5.40 (1.49) 90.3%	5.14 (1.79) 88.0%
Blame (Fred)	2.61 (0.88) 95.5%	2.73 (0.66) 98.4%	2.57 (1.00) 94.6%
Free Will (Fred)	2.54 (1.04) 93.3%	2.66 (0.89) 95.2%	2.48 (1.15) 92.4%
Moral responsibility (Arthur)	4.82 (1.91) 81.6%	4.97 (1.80) 83.9%	4.61 (2.11) 77.2%
Praise (Arthur)	2.01 (1.31) 80.4%	2.05 (1.25) 79.0%	1.94 (1.37) 81.5%
Free will (Arthur)	2.47 (1.16) 91.6%	2.55 (1.05) 91.9%	2.40 (1.28) 91.3%
Could have done otherwise (Fred)	88.3%	90.3%	84.8%
Bypassing	− 0.83 (1.57) 25.1%	− 0.99 (1.70) 24.2%	− 0.68 (1.53) 28.3%
Fatalism	0.11 (1.46) 49.2%	0.30 (1.39) 54.8%	0.07 (1.56) 47.8%
Intrusion	0.40 (1.55) 57.0%	0.62 (1.33) 62.9%	0.35 (1.67) 55.4%
Determinism	0.80 (1.53) 69.3%	0.77 (1.51) 74.2%	0.84 (1.59) 65.2%
*N*	179	62	92

% indicate the percentage of participants who gave an answer superior to the midpoint

As can be seen in Table 9, 88.3% of the remaining participants answered that Fred could have chosen not to steal John’s slice of cheesecake. Among those participants, 58.2% chose the first “conditional” interpretation of “could have done otherwise” (“if Fred’s desires, beliefs and/or character had been different, it might have happened that Fred chose not to steal the slice of cupcake”), while 13.3% chose the second “conditional” interpretation (“if what happened in the past had been different, it might have happened that Fred chose not to steal the slice of cupcake”), and 19.6% chose the “unconditional” interpretation. 8.9% answered “something else”.

Finally, we examined participants’ answers to the Bypassing, Fatalism, Intrusion, and Determinism questions. Results are presented in Table 9. As one can see, Bypassing scores were low, but Fatalism and Intrusion scores were still high. However, Determinism scores were high and above the midpoint, which suggests that participants understood that John’s universe is deterministic. This suggests that Intrusion measures do not properly measure the unconditional ability to do otherwise. Indeed, if they were, we should expect to observe a strong negative correlation between Intrusion and Determinism measures, but we found only a weak, non-significant correlation (*r* = − 0.13, *p* = 0.09). This means that our participants understood that John’s universe was deterministic, but did not involve bypassing. The only comprehension error remaining high was Fatalism.

To determine which comprehension errors drove participants’ judgments, we ran six multiple regression analyses with all three types of errors (Bypassing, Fatalism, and Intrusion) and Determinism scores as predictors and participants’ answers to the moral responsibility, blame/praise, and free will questions as dependent variables. Results are presented in Table 10. Overall, only confusion with bypassing seemed to impact participants’ judgments.

**Table 10:** Standardized coefficients and p-values for six multiple regression analyses with Bypassing, Fatalism, Intrusion and Determinism scores as predictors, and Moral responsibility, Blame/Praise, and Free will scores as dependent variables (Study 5)

	Bypassing	Fatalism	Intrusion	Determinism	*R*^2^
Moral responsibility (Fred)	β = − 0.15 p = .09	β = 0.06 p = 49	β = 0.15 p = .07	β = 0.15 p = .46	0.06*
Blame (Fred)	β = − 0.26 p = .005**	β = 0.12 p = .18	β = 0.10 p = .21	β = − 0.09 p = .24	0.08**
Free Will (Fred)	β = − 0.35 p < .001***	β = 0.14 p = .68	β = 0.11 p = .15	β = − 0.11 p = .15	0.14***
Moral responsibility (Arthur)	β = − 0.11 p = .21	β = − 0.14 p = .13	β = − 0.04 p = .65	β = 0.06 p = .41	0.04
Praise (Arthur)	β = − 0.13 p = .17	β = − 0.09 p = .33	β = 0.00 p = .97	β = 0.08 p = .29	0.04
Free Will (Arthur)	β = − 0.40 p < .001***	β = 0.13 p = .14	β = 0.08 p = .32	β = − 0.03 p = .70	0.15***

## Conclusion

Through five studies, we investigated folk intuitions about the free will and moral responsibility of people going through several repetitions of the same sequence of events (i.e. time loops).

First, we found that people did not seem to have any trouble imagining that the same events (including human actions) would repeat themselves again and again each time the time loop is reset. In all five studies, a wide majority of participants spontaneously answered that the same events would replay every time (in Study 1), or in 100 loops out of 100 (in Studies 2 to 4), or that the same events would necessarily happen once the day is rewound (Study 5). This suggests that their possible commitments to an indeterministic conception of human agency did not intrude and prevent them from imagining such scenarios—even when they were not explicitly asked to.

Second, participants did not seem to think that whether or not the same action would replay again and again was relevant to free will and moral responsibility. In Study 1, we found no difference in free will and moral responsibility ascriptions when participants were directly asked to compare agents in a deterministic time loop to agents in an indeterministic time loop. In Studies 2 and 3, participants who answered that the same behavior would repeat in 100 loops out of 100 still attributed free will and moral responsibility to agents in that loop. Moreover, whether they initially answered that the same behavior would repeat in 100 loops out of 100 did not significantly impact their answers to the moral responsibility, blame and punishment questions (*p* > 0.05) and only impacted answers to the free will question in Study 2.^4^ Finally, in Studies 4 and 5, even when participants were explicitly presented with a world in which the same behaviors repeated themselves in 100 loops out of 100 because “the same causes always have the same effects”, most participants still ascribed moral responsibility, blame, punishment and free will to agents. Taken together, these data suggest that participants do not see determinism as a threat to free will and moral responsibility, once it is presented in a setting that makes clear that determinism does not stem from forces that bypass one’s desires and reasons.

But were we truly successful in reducing participants’ comprehension errors? The results of Studies 4 and 5 suggest that few participants confused determinism with bypassing in our vignettes (only 8.3% in Study 4, up to 25.1% in Study 5), which is great since confusion with bypassing still led participants to attribute less free will and moral responsibility (see Tables 7 and 10). Confusion with fatalism was more frequent (around 25% in Study 4 and around 50% in Study 5) but this confusion had no significant effect on participants’ answers (which is coherent with previous results, see Andow & Cova, 2016). Overall, our setting did prevent most participants from confusing determinism with other constructs that threaten free will and moral responsibility.

However, things get more complicated when it comes to intrusion. The fact that most participants did not have trouble answering that the same behavior would repeat in 100 loops out of 100 suggests that their beliefs about indeterministic agency did not intrude with their interpretation of our scenarios. But, in Studies 2, 4 and 5, a vast majority of our participants answered that agents in the timeloop “could have chosen otherwise”. Additionally, a majority of our participants obtained scores above the midpoint on Nadelhoffer and colleagues’ measures of Intrusion in Studies 4 and 5. We thus have contradictory results: why do people seem to attribute indeterministic agency to agents when asked whether the agents could have done or chose otherwise, but not when asked whether the agents would behave in the same way in all 100 loops?

It could be that participants’ beliefs in indeterministic agency intrude only when they are asked about agents’ ability to do or choose otherwise, but not when they are asked whether agents will act in the same way in each repetition of the loop. However, there is no clear reason why this would be the case. An alternate hypothesis is thus that questions about how the agents could have done otherwise and Intrusion measures do not (only) measure the indeterministic, unconditional ability to do otherwise, but (also) the conditional ability to do otherwise. In Studies 4 and 5, we directly asked participants about their interpretation of “could have done otherwise” and we observed that a majority (55.4% in Study 4 and 71.5% in Study 5) chose the “conditional” definition. Moreover, even though those who chose the “unconditional” definition obtained higher intrusion scores (Study 4: *M* = 1.87, Study 5: *M* = 0.94), participants who chose the “conditional” definition still endorsed Intrusion measures (Study 4: *M* = 0.88, 71.7% of answers above midpoint; Study 5: 0.49, 58.4% of answers above midpoint). Overall, this suggests that the discrepancy between our questions about time loops and Intrusion measures might simply come from the fact that Intrusion measures fail to capture what they were intended to capture (the unconditional ability to do otherwise).

There are several reasons to be wary of the idea that Intrusion measures aptly capture perceptions of indeterministic agency. The first is that Intrusion measures contain expressions such as “could” and “being open for X to” that are ambiguous because they can be interpreted in both conditional and unconditional ways, while there seems to be no such ambiguity in our measure that only asks participants to give a number. The second is that, to our knowledge, the external validity of Intrusion measures has never been assessed. Nadelhoffer and colleagues (in press) made the brilliant effort of having expert philosophers assess the validity of their measure, but non-experts might sometimes interpret items in a way unintended by philosophers (see Cova, forthcoming-b for an example). So, there is no independent reason to think that Intrusion measures actually measure the unconditional ability to do otherwise rather than the conditional one.

To assess the validity of Intrusion measures, we introduced in Study 5 a measure of participants’ perception of Determinism, built from the items of the Determinism subscale of the Free Will Inventory, a validated measure of belief in Determinism. If Intrusion measures truly measured perceptions of the unconditional ability to do otherwise, which is incompatible with Determinism, we should expect to find a substantial negative correlation between the two measures. However, we found no significant correlation between the two. Of course, it is possible that the Determinism subscale is not a good measure of participants’ perceptions of Determinism, but this is one more reason to doubt the validity of Intrusion measures. Rather, participants’ answers to the Determinism subscale and to the question about whether the same behavior would replay in 100 loops out of 100 seem to suggest that participants were able to understand that the universe we depicted in Study 5 was deterministic.

Finally, it should be noted that *if* Intrusion measures actually measured people’s attribution of the unconditional ability to do otherwise, then this would mean that we can take our results to show that this ability is mostly irrelevant to the folk attributions of moral responsibility. As can be seen in Tables 8 and 10, participants’ scores on the Intrusion measures did not significantly predict their ascriptions of moral responsibility, blame, and punishment. Moreover, it only weakly predicted attributions of free will, and only for Study 4. Even if we only keep participants who obtained a total Intrusion score inferior to the midpoint, we find that 73.7% attributed moral responsibility, 90.2% attributed blame, 90.2% attributed punishment, and 95.1% attributed free will in Study 4, and that 81.1% attributed moral responsibility, 90.6% attributed blame, and 88.7% attributed free will in Study 5. Thus, it cannot be said that our participants attribute free will and moral responsibility *because* they attribute agents the unconditional ability to do otherwise.

Overall, the errors identified by Nadelhoffer and colleagues only explain between 7 and 16% of the variance in our participants’ answers (see Tables 7 and 10). This means that we succeeded in producing vignettes in which participants’ intuitions about free will and moral responsibility are not mainly driven by such confusions. Our results suggest that most people did not see free will and moral responsibility to be incompatible with determinism, when determinism is presented through time loops. As such, we take our results to provide additional reasons to think that most people are natural compatibilists.

### Supplementary Information

Below is the link to the electronic supplementary material.

Supplementary file1 (DOCX 8 KB)

## Data Availability

All materials and data are publicly available at osf.io/hm7ze/ (https://doi.org/10.17605/OSF.IO/HM7ZE).

## Footnotes

1 Rose and Nichols (2013) have objected to Murray and Nahmias’ conclusion that mediation models in which free will and moral responsibility judgments are mediators rather than bypassing scores are a better fit of the data. For the problems raised by the (over)interpretation of model fits, see Cova (forthcoming-a).

2 This experimental nihilism is premised on the idea that there is no worthy alternative to vignettes. However, one might argue that people’s intuitions about free will and determinism can be explored using scales. For example, Nadelhoffer and colleagues (2014) designed two independent measures of belief in free will and belief in determinism. However, both measures tend to be orthogonal, meaning that they tend not to correlate (either positively or negatively) with determinism. This provides reason to think that folk are natural compatibilists, unless one extends experimental nihilism to the validity of these scales.

3 We thank Samar Haidar for her suggestion that people might react differently to a case presented as a ‘rewind’ compared to a case presented as a ‘loop’.

4 Contrary to what might be expected, in Study 4, free will ratings were higher when participants answered that the same behavior would occur in 100 loops out of 100. This might point to the fact that participants see randomness and luck as a more serious threat to free will than determinism.
