# Supplementary material for: “One more time”: time loops as a tool to investigate folk conceptions of moral responsibility and human agency
Source: Synthese. 2023 Aug 29;202(3):83. doi: 10.1007/s11229-023-04245-9 (PMC10465374; doi:10.1007/s11229-023-04245-9)
Supplement: Supplementary file 1 — Supplementary file1 (DOCX 8 KB) [file 11229_2023_4245_MOESM1_ESM.docx]

**“One more time”: Time loops as a tool to investigate folk conceptions of moral responsibility and human agency**

**Supplementary Materials**

*A. Qualitative analysis for Study 3*

To check whether participants understood the case and questions they were presented with, the second author on this paper was tasked with coding participants’ justifications to the Moral Responsibility question. Coding categories are presented in Table S1.

| *Coding categories for Study 3* | | |
| --- | --- | --- |
| DID | Participants emphasize that Al was the one who did it. | 12.7% |
| CHOICE | Participants emphasize that Al had the choice. | 34.5% |
| NO CHOICE | Participants emphasize that Al had *no* choice. | 4.4% |
| INTENTION | Participants emphasize that Al had the intention to kill Bob or that he did it intentionally. | 0.9% |
| PERSONAL INTEREST (+) | Participants emphasize the fact that Al had personal interests in killing Bob, to *increase* his responsibility. | 4.8% |
| PERSONAL INTEREST (-) | Participants emphasize the fact that Al had personal interests in killing Bob, to *decrease* his responsibility. | 1.7% |
| FATALISM | Participants endorse a fatalistic interpretation of the time loop, leading them to attribute *less* moral responsibility. | 0.9% |
| NO FATALISM | Participants stress the fact that, despite the weight of external circumstances, Al was not fated to kill Al and was the one choosing to do it, leading them to attribute *more* moral responsibility. | 5.7% |
| HESITANT | Participants grant Al extenuating circumstances but still deem him morally responsible. | 1.3% |
| MORALIZATION | Participants emphasizes the moral wrongness of Al’s action. | 3.5% |
| ERROR | Participants failed to understand some aspect of the vignette or the question. | 21.4% |
| *ERROR_JOHN | Subcategory of ERROR: participants answered as if the moral responsibility question was about John. | 16.2% |
| NO JUSTIFICATION | Participants gave no justification. | 7.4% |

**Table S1.** Coding categories for Study 3 (Justifications).

We excluded participants whose justification fell in the FATALISM, ERROR or NO JUSTIFICATION category. We were left with 167 participants. Of those, 83.2% answered that Brett would solve the math theorem 100 times out of 100, and 80.2% answered that Al would shoot Bob in 100 loops out of 100. Keeping only participants who answered that Al would shoot Bob in 100 loops out of 100, we found that 91.8% of participants attributed moral responsibility, 97.0% attributed blame, 97.0% attributed punishment, and 93.3% attributed free will. Overall, exclusion did not change our results.
